# Application of industrial treatments to donor human milk: influence of pasteurization treatments, storage temperature, and time on human milk gangliosides

**DOI:** 10.1038/s41538-018-0013-9

**Published:** 2018-03-13

**Authors:** Jaime Salcedo, Sercan Karav, Annabelle Le Parc, Joshua L. Cohen, Juliana M. L. N. de Moura Bell, Adam Sun, Matthew C. Lange, Daniela Barile

**Affiliations:** 1 Department of Food Science and Technology, University of California Davis, Davis, CA 95616 USA; 2 Department of Molecular Biology and Genetics, Mart University, Canakkale, Turkey; 3 Prolacta Bioscience, City of Industry, Los Angeles, CA 91746 USA; 4 Foods for Health Institute, University of California Davis, Davis, CA 95616 USA; 5 Present Address: Prolacta Bioscience, City of Industry, Los Angeles, CA USA

**Keywords:** Glycobiology, Nutrition

## Abstract

Donor milk is the best option when mother’s own milk is unavailable. Heat treatments are applied to ensure donor milk safety. The effects of heat treatments on milk gangliosides—bioactive compounds with beneficial antibacterial, anti-inflammatory, and prebiotic roles—have not been studied. The most abundant gangliosides in non-homogenized human milk were characterized and quantified by liquid chromatography–mass spectrometry (LC–MS)/MS before and after pasteurization treatments mimicking industrial conditions (63 °C/30 min, 72 °C/15 s, 127 °C/5 s, and 140 °C/6 s). Ganglioside stability over a 3-month period was assessed following the storage at 4 and 23 °C. Independent of the heat treatment applied, gangliosides were stable after 3 months of storage at 4 or 23 °C, with only minor variations in individual ganglioside structures. These findings will help to define the ideal processing and storage conditions for donor milk to maximize the preservation of the structure of bioactive compounds to enhance the health of fragile newborns. Moreover, these results highlight the need for, and provide a basis for, a standardized language enabling biological and food companies, regulatory agencies, and other food stakeholders to both annotate and compute the ways in which production, processing, and storage conditions alter or maintain the nutritive, bioactive, and organoleptic properties of ingredients and foods, as well as the qualitative effects these foods and ingredients may have on conferring phenotype in the consuming organism.

## Donor milk treatment: key nutrients preserved after pasteurization

Donor human milk, the best alternative to mother’s own milk, usually needs to be pasteurized before use out of safety concerns. Daniela Barile at University of California Davis, USA, and colleagues studied the effects of heat treatment and storage temperature and time on milk gangliosides, a class of sugar-derived compounds important for neural and brain development of newborns, among other bioactivities. They found that, while there were minor structural changes during mimicked industrial pasteurization processes, gangliosides remain stable for at least three months either in the refrigerator or at room temperature. These results may help standardize the processing protocols and storage conditions for donor milk, and the methods can be extended to other bioactive components.

## Introduction

Breastfeeding is generally considered the optimal infant nutrition for the first 6 months of life. However, if breastfeeding is not possible, international authorities (World Health Organization (WHO), American Academy of Pediatrics, European Society of Pediatric Gastroenterology, Hepatology and Nutrition) recommend using pasteurized donor breast milk as the most adequate alternative.^1^ Despite the efforts made to humanize the infant formula, its composition lacks many of the immunological and protective factors—oligosaccharides and glycolipids, among others—present in human milk.^2,3^ Characterization and quantification of the bioactive components found in milks, together with the characterization and quantification of the processing conditions that alter them, provide baseline profiles for formula makers to mimic the nutrient and bioactive properties found in human milk.

Donor milk affords benefits beyond simple provision of nutrients for newborns, including protection from infection, lower incidence of necrotizing enterocolitis, improved food tolerance, and better long-term neurological development.^4–7^ The main concern regarding donor milk is its microbiological safety, as preterm infants are particularly susceptible to bacterial infections. Current official protocols include pasteurization and freezer storage at –20 °C to eliminate hazards for newborns and preserve bioactive compounds. Several studies evaluated the effect of heat treatment and storage conditions on human milk components: Holder pasteurization (63 °C/30 min) and conventional pasteurization (75 °C/15 s) do not increase lipid oxidation, but they do diminish glutathione oxidase and glutathione peroxidase activities, therefore, decreasing the total antioxidant potential in the milk.^8^ The literature reports conflicting results regarding the effects of storage conditions on milk compounds. Some authors reported that fatty acids are stable at room temperature (RT), 4, –20, or –80 °C,^9^ for 12 months. Additionally, oxidative status and lipase activity are unchanged during the storage at –20 °C. However, others reported a decrease in total fat content, lactose, and oligosaccharides overtime in pasteurized milk stored at –20 °C.^10^ Not only does milk composition vary depending on the heat treatment or storage conditions, but its biological properties are also modified. Previous works reported that, compared to untreated milk, the bactericidal activity of human milk against *Escherichia coli* decreased significantly, when using the conventional treatments employed by milk banks.^11^ Decreases in bactericidal activity were also observed in non-pasteurized milk after 3 months storage at –20 and –80 °C^12^ but not after 1 month at –80 °C.

Although, human milk oligosaccharides have gained importance as new bioactive compounds,^13^ other glycoconjugates, such as glycolipids, which may contribute to breast milk protective functions, have been neglected. Recent publications reported that in the newborn, glycolipids play key roles in development, such as brain formation, immune response maturation, and protection from allergy.^7,14–17^ Human milk contains glycolipids primarily as gangliosides almost exclusively associated with the milk fat globule membrane.^18^ These gangliosides can be defined as glycosphingolipids, as they are composed of a sphingoid base linked to a fatty acid by an amide linkage (ceramide) bound to an oligosaccharide chain of variable size containing one or more sialic acid moieties.^18^ The possible combinations of the constituent monosaccharides, sialic acid molecules, and ceramide structures give rise to countless types of gangliosides varying in composition, structure, and linkages. Thus, gangliosides vary widely in both content and profile in milk during lactation.

The total ganglioside content is at its highest in colostrum, with an average value of 11 mg/L lipid-bound sialic acid, and its concentration decreases during lactation until it becomes barely detectable in mature human milk (0.3 mg/L lipid-bound sialic acid).^3^ During the first few days of lactation, GD3 (Neu5Ac α2-8 Neu5Ac α2-3 Gal β1-4Glc β1-1 ceramide) is the most abundant ganglioside in human milk, comprising almost 65% of total gangliosides, followed by GM3 (Neu5Ac α2-3 Gal β1-4Glc β1-1 ceramide, 25% of the total). As lactation advances, their relative abundance reverses, with GM3 being the most abundant in mature milk (85% of total gangliosides) followed by GD3 (9%). Throughout the entire lactation period, other gangliosides are detected in lower concentration: *O*-acetyl GD3, GM2 (GalNAcβ1-4(Neu5Ac α2-3)Galβ1-4Glc β1-1 ceramide), and GM1 (Galβ1-3GalNAcβ1-4[Neu5Ac α2-3] Galβ1-4Glcβ1-1 ceramide).^3^

Due to the low content of gangliosides (ppm level) and to the high-structural diversity of those compounds, the development of a fast and accurate methodology for their determination is challenging. The classic methodology for quantifying the total amount of gangliosides, based on the resorcinol-HCl reagent and high-performance thin-layer chromatography coupled to densitometric detection^19^ is laborious, time-consuming, and does not allow the full elucidation of their structures.^18^ Recently, our group developed a method to identify and quantify gangliosides in human and bovine milk by LC–MS/MS and ultrahigh-performance liquid chromatography-quadrupole time-of-flight (UHPLC-qTOF).^20,21^ With the introduction of MS techniques, it is possible not only to characterize both glycan and ceramide lipid portions, but also to accurately quantitate each ganglioside.^21^

As studies evaluating the effects of heat treatments on human milk ganglioside content and profile are lacking, the present work was aimed at the identification and quantitation of the structural changes of the main gangliosides in human milk after being subjected to different pasteurization treatments and determination of their stability under different storage conditions. This study will help to define the ideal processing and storage conditions for donor milk to preserve the structure of bioactive compounds for enhancing the health of fragile newborns.

## Results

The concentration of GM3, GD3, and total gangliosides in the non-treated human milk is 9.06, 5.66, and 14.72 mg/L, respectively. The direct effect of each heat treatment, evaluated by measuring the glycolipid concentration just after heat treatment, is shown in Table 1. Only the concentration of GM3 statistically increases when human milk is subjected to HT-2, rest of the conditions are not showing any effect on the compounds analyzed. During the 90 days study, the total ganglioside content, considered as the sum of GM3 and GD3, ranged between 14.40 and 15.55 mg/L (Table 2), while individual GM3 and GD3 contents varied between 8.83 and 9.69 mg/L, and 5.47 and 5.86 mg/L for GM3 and GD3, respectively.

**Table 1:** Concentration of GM3, GD3, and total gangliosides in human milk subjected or not to different heat treatments at day 0 posttreatment

	No heat treatment	HT-1	HT-2	HT-3	HT-4
[GM3] (mg/L)	9.05 ± 0.13	9.25 ± 0.36	9.50 ± 0.11*	9.25 ± 0.21	9.30 ± 0.14
[GD3] (mg/L)	5.66 ± 0.24	5.70 ± 0.41	5.84 ± 0.33	5.69 ± 0.29	5.76 ± 0.43
Total GG (mg/L)	14.72 ± 0.15	14.94 ± 0.76	15.34 ± 0.27	14.94 ± 0.29	15.06 ± 0.44

Results expressed as mg ganglioside/L human milk (*n* = 3). Results expressed as mean ± standard deviation (*n* = 3)

*HT-1* Holder pasteurization (63 °C/30 min), *HT-2* standard high-temperature-short-time pasteurization (72 °C/15 s), *HT-3* extended shelf-life protocol (127 °C/5 s), *HT-4* ultrahigh-temperature pasteurization (140 °C/6 s)

* Indicates statistical differences with no heat-treated sample (Tukey’s test) (*p* < 0.05)

**Table 2:** Concentration of total gangliosides (sum of GM3 and GD3) in human milk subjected to different heat treatments and storage times

	[Total gangliosides] (mg/L)									
Storage time	No heat treatment		HT-1		HT-2		HT-3		HT-4	
	Room temperature (23 °C/40% RH)	Cold temperature (4 °C)	Room temperature (23 °C/40% RH)	Cold temperature (4 °C)	Room temperature (23 °C/40% RH)	Cold temperature (4 °C)	Room temperature (23 °C/40% RH)	Cold temperature (4 °C)	Room temperature (23 °C/40% RH)	Cold temperature (4 °C)
Day 7	14.66 ± 0.79	14.56 ± 0.74	14.69 ± 0.43	14.69 ± 0.57	15.35 ± 0.78	15.38 ± 0.96	14.85 ± 0.36	14.98 ± 1.34	14.95 ± 0.38	14.96 ± 0.94
Day 30	14.51 ± 0.175	14.59 ± 0.60	14.43 ± 0.67	14.69 ± 0.51	15.21 ± 0.38	15.33 ± 0.37	14.93 ± 0.80	14.97 ± 0.44	15.27 ± 0.30	15.37 ± 0.67
Day 60	14.46 ± 0.468	14.72 ± 0.85	14.60 ± 0.17	14.62 ± 0.10	15.43 ± 0.64	15.44 ± 0.58	14.85 ± 0.29	15.00 ± 0.34	15.03 ± 0.13	15.14 ± 0.66
Day 90	14.40 ± 0.489	14.69 ± 0.25	14.61 ± 0.39	14.66 ± 0.15	15.17 ± 0.58	15.55 ± 0.75	14.84 ± 0.83	14.94 ± 0.17	14.85 ± 0.47	15.08 ± 0.74

Results expressed as mg total ganglioside/L human milk (*n *= 3). Results expressed as mean ± standard deviation (*n* = 3)

*HT-1* Holder pasteurization (63 °C/30 min), *HT-2* standard high-temperature-short-time pasteurization (72 °C/15 s), *HT-3* extended shelf-life protocol (127 °C/5 s), *HT-4* ultrahigh-temperature pasteurization (140 °C/6 s), *RH* relative humidity

**Fig. 1:**
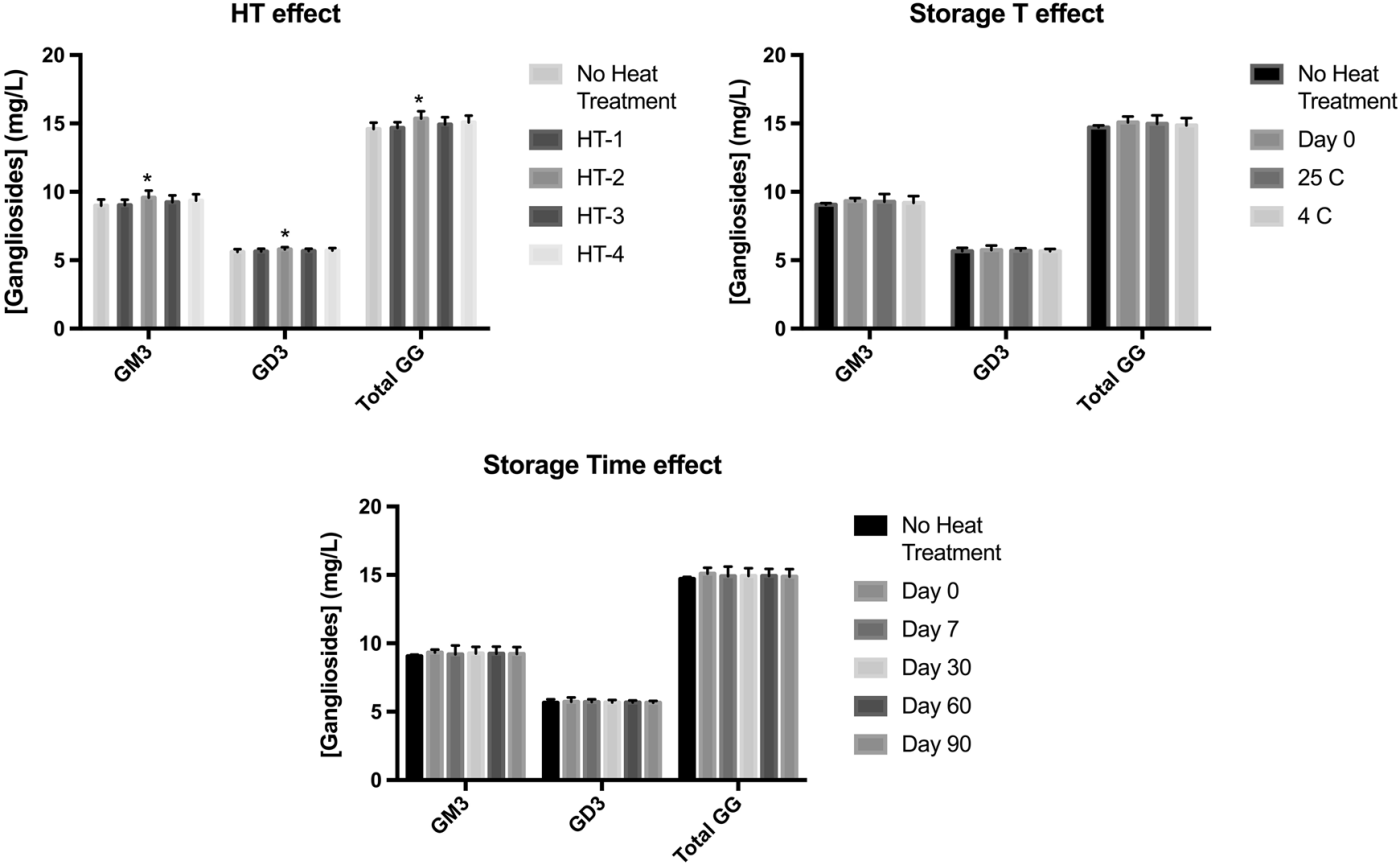
Effect of heat treatment and storage time on human milk gangliosides (GM3, GD3, Total, GG). Multiple analysis of variance using HSD-Tukey was used as a post hoc analysis test (*p < *0.05). Results are expressed as mean ± standard deviation (*n* = 3). * Indicate statistical differences with non-treated milk No heat treatment: pooled human milk sample not subjected to any heat treatment

When the effect of the different heat treatment over gangliosides concentration during 90 days was assessed at two storage conditions (Tables 2 and 3), neither time nor temperature significantly affected ganglioside content during the 90 days of treatment (Fig. 1). However, heat treatment significantly altered both total and individual ganglioside contents. HT-2 increased the content of GM3, GD3, and total gangliosides compared with unheated milk (Fig. 1).

**Table 3:** Concentration of GM3 and GD3 in human milk subjected to different heat treatments and storage times

Storage time	No heat treatment		HT-1		HT-2		HT-3		HT-4	
	Room temperature (23 °C/40% RH)	Cold temperature (4 °C)	Room temperature (23 °C/40% RH)	Cold temperature (4 °C)	Room temperature (23 °C/40% RH)	Cold temperature (4 °C)	Room temperature (23 °C/40% RH)	Cold temperature (4 °C)	Room temperature (23 °C/40% RH)	Cold temperature (4 °C)
[GM3] (mg/L)										
Day 7	8.96 ± 0.52	8.97 ± 0.72	9.05 ± 0.33	9.07 ± 0.76	9.58 ± 0.95	9.55 ± 0.80	9.14 ± 0.28	9.25 ± 1.23	9.19 ± 0.29	9.20 ± 0.79
Day 30	8.94 ± 0.25	8.96 ± 0.66	8.83 ± 0.85	9.06 ± 0.23	9.45 ± 0.36	9.60 ± 0.14	9.27 ± 0.67	9.28 ± 0.300	9.52 ± 0.017	9.62 ± 0.68
Day 60	8.91 ± 0.35	9.08 ± 0.91	8.95 ± 0.04	8.96 ± 0.08	9.69 ± 0.76	9.65 ± 0.64	9.14 ± 0.05	9.25 ±0.52	9.34 ± 0.23	9.45 ± 0.74
Day 90	8.93 ± 0.53	9.10 ± 0.21	8.98 ± 0.38	9.00 ± 0.17	9.41 ± 0.64	9.69 ± 0.66	9.18 ± 0.88	9.26 ± 0.24	9.28 ± 0.58	9.40 ± 0.65
[GD3] (mg/L)										
Day 7	5.71 ± 0.38	5.59 ± 0.23	5.64 ± 0.12	5.62 ± 0.19	5.77 ± 0.21	5.83 ± 0.30	5.65 ±0.17	5.73 ± 0.12	5.76 ± 0.10	5.78 ± 0.19
Day 30	5.57 ± 0.17	5.63 ± 0.33	5.60 ± 0.29	5.63 ± 0.29	5.76 ± 0.04	5.73 ± 0.24	5.66 ±0.18	5.69 ± 0.14	5.75 ± 0.15	5.75 ± 0.12
Day 60	5.554 ± 0.19	5.64 ± 0.14	5.65 ± 0.17	5.66 ± 0.10	5.74 ± 0.13	5.79 ± 0.15	5.65 ±0.25	5.75 ± 0.18	5.68 ± 0.14	5.70 ± 0.14
Day 90	5.472 ± 0.10	5.59 ± 0.13	5.63 ± 0.08	5.66 ± 0.02	5.76 ± 0.07	5.86 ± 0.12	5.65 ± 0.22	5.67 ± 0.12	5.57 ± 0.13	5.68 ± 0.14

Results expressed as mg ganglioside/L human milk (*n* = 3). Results expressed as mean ± standard deviation (*n* = 3)

*HT-1* Holder pasteurization (63 °C/30 min), *HT-2* standard high-temperature-short-time pasteurization (72 °C/15 s), *HT-3* extended shelf-life protocol (127 °C/5 s), *HT-4*, ultrahigh-temperature pasteurization (140 °C/6 s), *RH* relative humidity

**Fig. 2:**
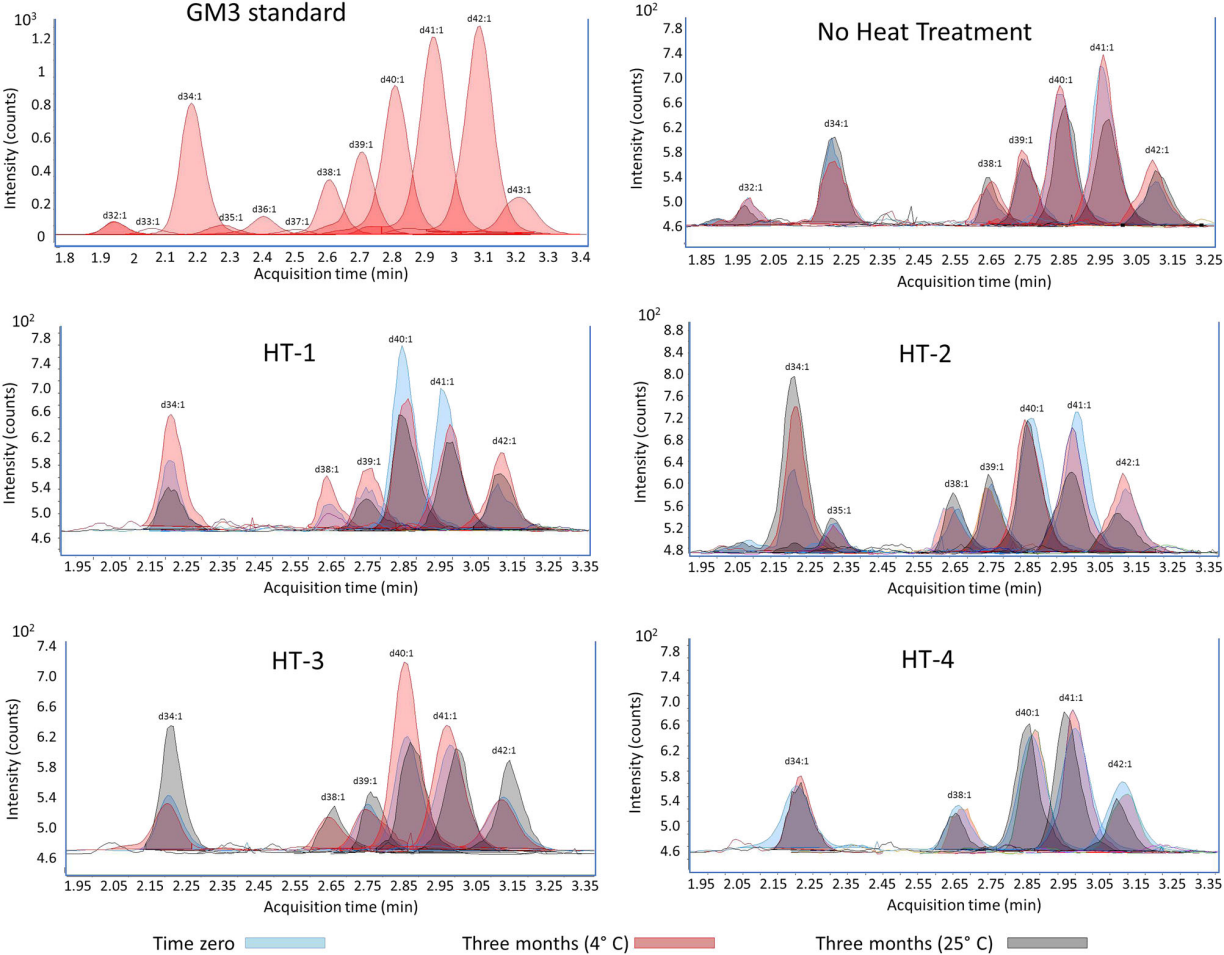
Variation of GM3 species before and after heat treatment (time zero), and after 3 months storage at 4 or 25 °C. A GM3 standard (0.1 mg/L) is also shown. HT-1 Holder pasteurization (63 °C/30 min), HT-2 standard high-temperature-short-time pasteurization (72 °C/15 s), HT-3 extended shelf-life protocol (127 °C/5 s), HT-4 ultrahigh-temperature pasteurization (140 °C/6 s). No heat treatment: pooled human milk sample not subjected to any heat treatment

**Fig. 3:**
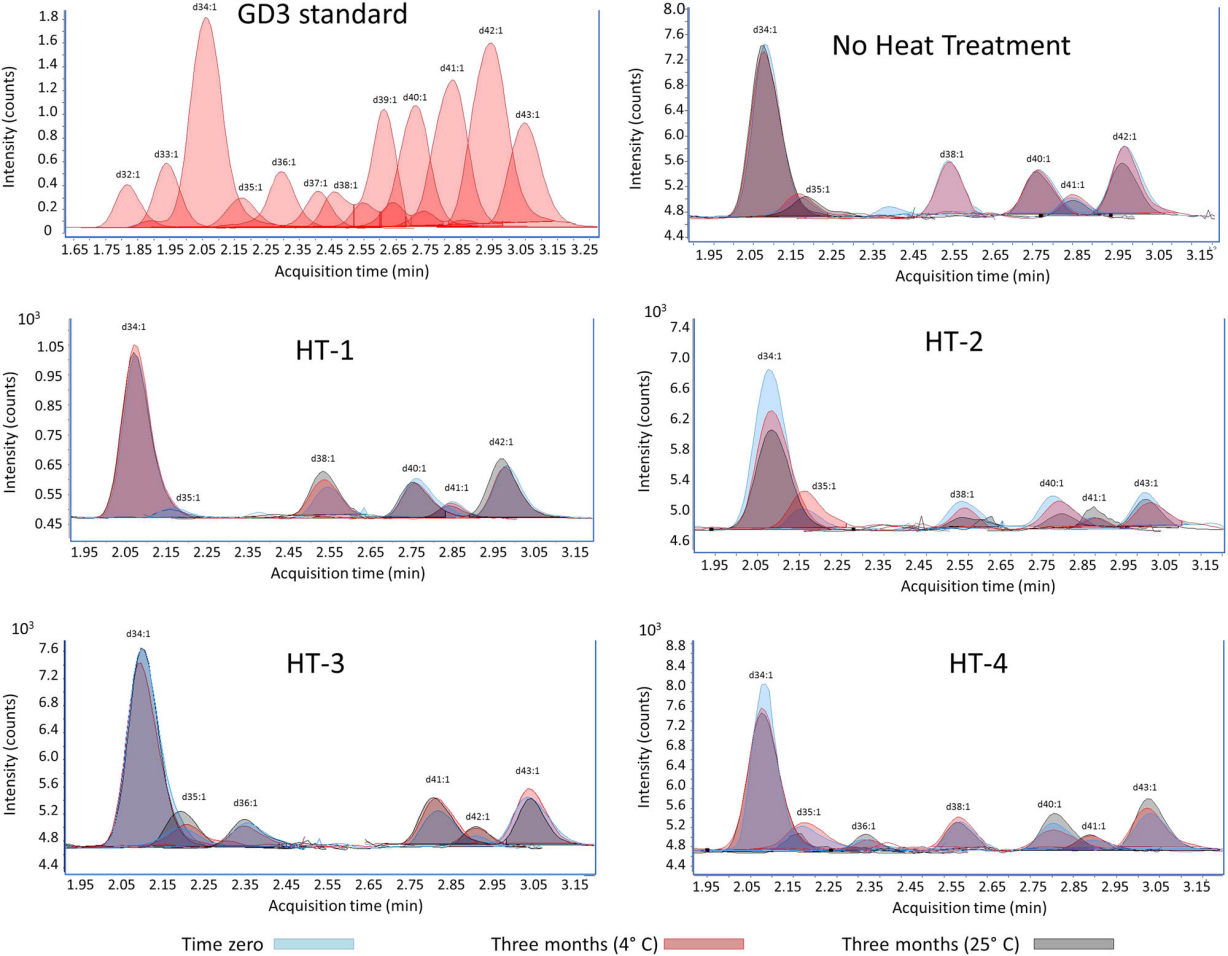
Variation of GD3 species before and after heat treatment (time zero), and after 3 months storage at 4 or 25 °C. A GM3 standard (0.1 mg/L) is also shown. HT-1 Holder pasteurization (63 °C/30 min), HT-2 standard high-temperature-short-time pasteurization (72 °C/15 s), HT-3 extended shelf-life protocol (127 °C/5 s), HT-4 ultrahigh-temperature pasteurization (140 °C/6 s). No heat treatment: pooled human milk sample not subjected to any heat treatment

Although, the variation in terms of content were not statistically significant during storage time, there was a qualitative shift in the ceramide component of individual gangliosides (Figs. 2 and 3). High-temperature treatments increased the formation of GD3(d35:1), GD3(d36:1), GM3(38:1), G3(d39:1), and GM3(43:1), whereas at lower temperatures the ganglioside profile was similar to that of unheated milk. During storage, there were minor variations in the gangliosides profile depending on the heat treatment and storage time. The ganglioside species profile of unheated milk remained stable when stored at 4 °C, but the GM3(d40:1) and GM3(d41:1) species were reduced when samples were stored at 23 °C. When milk was heat treated, ganglioside GD3 was more stable than GM3 after 3 months of storage, the GD3(d34:1) species decreased when milk was subjected to HT-2 (HTST). This decrease in GD3 (d34:1) species became greater when the sample was stored at 23 °C (Fig. 2b). In contrast, the effect of storage time on the GM3 species was more variable than temperature effects, GM3(d34:1), GM3(d40:1), and GM3(d41:1) were the most affected (Fig. 3a). It is noteworthy that there were minimal variations in milk GM3 and GD3 over storage time independent of storage temperature. HM subjected to HT-4 showed minimal variations in both the GM3 and GD3 species during storage independent of storage conditions.

## Discussion

The present work aims to evaluate if the glycolipid content in non-homogenized human milk remain stable overtime independently of storage and temperature conditions (i.e., during 90 days refrigerated at 4 °C or stored at RT). Furthermore, the four heat treatments most commonly used by the milk banks and by the dairy industry (Holder pasteurization, high-temperature-short-time pasteurization (HTST), extended shelf-life (ESL), and ultrahigh-temperature (UHT)) were evaluated to study their suitability for human milk processing in terms of glycolipid content and stability.

Human milk glycolipids content is known to vary widely depending on the lactation stage, diet, and ethnicity, with reported concentrations for GM3 and GD3 ranging from 2.8 mg/L to 14.3 mg/L for colostrum, 0.9 mg/L to 25.1 mg/L for transitional milk and 1.6 mg/L to 23.8 mg/L and mature milk, respectively.^22–24^ The total glycolipid content of the HM pool used in our study ranged from 14.72 mg/L at time zero to 14.40 mg/L after 60 days at RT. These values are in the middle-range of those reported for mature HM. Individual GD3 and GM3 content (8.83–9.69 mg/L and 5.47–5.86 mg/L, respectively, for time zero and after 60 days at RT and trends in our study reflected the published data, with GM3 being the dominant species and GD3 showing lower values. Similarly, we found that none of the heat treatments significantly altered the content of individual or total gangliosides, with the exception of HT-2, where a statistically significant increase in GM3 was observed. The evaluation of the effect of heat treatment and storage temperature showed that neither significantly affected ganglioside content during 90 days of treatment (Fig. 1). However, heat treatment caused the changes in both total and individual ganglioside contents. HT-2 increased the content of GM3, GD3, and total gangliosides compared with unheated milk, and HT-4 increased total gangliosides compared with unheated milk (Fig. 1).

Ceramides are the core structure of sphingolipids, which are ubiquitous components of eukaryotic cell membranes and play several roles in the human body: apoptosis, neuronal development and membrane formation and stabilization.^25^ Classically, gangliosides are considered biologically active because of their oligosaccharide chain. However, nowadays the ceramide moiety is gaining more attention thanks to recent studies demonstrating that glycolipids’ effects are not only depending on their oligosaccharide chain, but also on the ceramide composition. As an example, Martín et al.^26^ reported that enterotoxigenic *E. coli* (ETEC) strains bind to gangliosides in a ceramide-dependent process and its adhesion properties changed with different sphingoid base and fatty acid composition; whereas, the oligosaccharide moiety remained unchanged. In another study, Ladisch et al.^27^ reported that monosialogangliosides with short and medium chain fatty acids (d18:1-C2:0-GM3 and d18:1-C14:0-GM3) were more immunosuppressive than those with long-chain fatty acids (d18:1-C18:0-GM3 and d18:1-C24:0-GM3). These studies suggest that glycolipid ceramides play an active role in their biological activity, although, scarce studies have been carried out for a deeper evaluation of their individual activity. Although, glycolipid content does not change during storage and is not affected by heat treatments, their molecular species shifted, varying the abundance of their ceramides (Figs. 2 and 3). These modifications may modulate their biological activity, and point to the need for more in-depth studies to evaluate the direction of these changes.

Milk gangliosides are almost totally contained within the milk fat globule membrane, with their ceramide component forming part of the lipid bilayer. In this study, there were no significant modifications in milk fat globule membrane content for gangliosides, likely due to the lack of homogenization, as that step is not part of the conventional processing of donor milk. Evidence suggests human milk homogenization can increase fat absorption and weight gain in preterm infants^28^ and drastically changes the organization of milk components and decreases the size of milk fat globules,^29^ possibly increasing gangliosides accessibility. Therefore, alterations to milk fat globule structure could lead to increased ganglioside release, rendering these compounds are more vulnerable to heat treatment with consequent modifications in their digestibility and/or bioavailability, as well as their biological activity. Considering these factors, future studies on human milk should consider the effect of biological effects of homogenization relative to its industrial application.

## Conclusions

This study evaluated the effects of different pasteurization conditions and storage conditions on the composition and quantity of the most abundant human milk gangliosides (GM3, GD3). Our results demonstrate that the ganglioside content of pasteurized human milk at 60 °C/30 min, and 127 °C for 5 s, remains stable up to 90 days when stored at either refrigerator or RT, even though minor structural changes occur depending on the specific heat treatment. This type of study, if extended to more classes of bioactive components, will be useful to characterize and define ideal processing and storage conditions for donor milk and help preserve the structure and functionality of these bioactive compounds, with the final goal of enhancing fragile newborn health. At the same time, broader and more standardized annotation of bioactive food components will provide a platform for computing the ways in which processing and storage conditions alter or maintain the nutritive, bioactive, and organoleptic properties of ingredients and foods, as well as the qualitative effects these foods and ingredients may have on conferring health phenotypes in the consuming organism. Follow-up work constructing standardized ontologies to capture this knowledge and make it queriable is imminent.

## Materials and methods

### Reagents

High-performance liquid chromatography (HPLC)/MS grade methanol, acetic acid, and isopropyl alcohol were purchased from Sigma (St. Louis, MO, USA), and certified ACS grade chloroform came from Fisher (Fair Lawn, NJ, USA). Ammonium acetate was of analytical reagent grade and was acquired from Merck (Darmstadt, Germany), and C8 cartridges were obtained from Supelco (Bellefonte, PA). The *N*-omega-CD3-octadecanoyl GM3 (IS), and GM3 and GD3 ganglioside standards from buttermilk were purchased from Matreya (Pleasant Gap, PA, USA). The stock solutions of both standards were prepared separately in methanol to obtain a concentration of 1 mg/mL. Milli-Q grade water was used for all experiments.

### Samples

Individual human milk samples from five healthy women between 90 and 180 days of lactation were collected using breast pumps, placed in sterile polypropylene containers, immediately frozen at –20 °C, and sent to Prolacta Bioscence, where they were stored at –30 °C. Once all samples for the study were collected, they were sent to the UC Davis facilities for analysis and processing. The sample temperature remained at –30 °C for a maximum 1 month until the experiments were carried out.

All donors gave written informed consent to use the donated milk for research purpose and the study was approved by the University of California-Davis Ethics Committee.

### Pasteurization treatments

Prior to pasteurization treatments, individual human milk samples were defrosted and pooled. The pooled sample was used as starting materials for all the treatments. Four different pasteurization treatments typically employed in industry were applied to pooled human milk at the UC Davis Milk Processing Lab using a UHT/HTST Lab 25 EHV hybrid continuous pasteurizer with touch screen control and a combined sterile product outlet/automatic filling control (SPO-AFC, MicroThermics, Raleigh, NC, USA). Heat treatment 1 (HT-1) conditions were those described for classical Holder pasteurization (63 °C for 30 min); heat treatment 2 (HT-2) consisted of a standard HTST and a holding temperature of 72 °C for 15 s; heat treatment 3 (HT-3) conditions resembled an ESL protocol, wherein milk was pasteurized for 5 s at 127 °C; heat treatment 4 (HT-4) was an UHT pasteurization (140 °C for 6 s). The heat-treated human milk was collected from the sterile product outlet in a high efficiency particulate air (HEPA)-filtered laminar flow cabinet into autoclaved containers. Each heat treatment was used to process 5 L of the human milk pool, with a result of 4 L of heat-treated milk being divided into aliquots of 14 mL for the storage study.

For each pasteurization treatment human milk aliquots were split into three groups: time zero, storage at RT, and cold storage (CS). The time-zero samples were processed on the same day as the heat treatment was carried out. RT samples were stored in a room with temperature and relative humidity (RH) fixed at 23 °C and 40%, respectively, while CS samples were stored at 4 °C. Samples were analyzed on days 0, 7, 30, 60, and 90 after heat treatment for both storage conditions (Suppl. Figure 1). Samples were stored away from direct light for the duration of the stability study.

### Glycolipid extraction

Samples were thawed and mixed in a water bath at 30 °C before ganglioside extractions. Gangliosides were extracted as described elsewhere with minor modifications.^21^ Briefly, gangliosides were separated from neutral lipids by two consecutive extractions: 2 mL of milk sample was mixed with methanol/chloroform/water (4:2:1 v/v/v), sonicated for 10 min, and centrifuged for 5 min at 8800×*g* at 4 °C. Two milliliters of water were added for phase separation and the aqueous phase was collected. The lipid phase was re-extracted with 11 mL of methanol/chloroform/water (2:1:0.7 v/v/v) following the same procedure. The lipid phase was extracted using 0.1 M KCl instead of water. The combined aqueous supernatants were dried in a SpeedVac rotor concentrator (Savant Instruments Inc., Holbrook, NY, USA) and the lyophilized sample was resuspended in 1 mL of the methanol/water (1/1 v/v) solution.

The ganglioside extract was purified by C8 solid-phase extraction (SPE).^21^ An SPE cartridge was conditioned with 5 mL of the methanol–water (1:1, v/v) solution, the sample was loaded and washed by 5 mL of the same methanol–water (1:1, v/v) solution, and gangliosides were eluted with 10 mL of isopropyl alcohol−methanol (1:1, v/v) solution. The ganglioside eluate was divided into aliquots of 500 μL, an internal standard (IS) was added to obtain a final concentration of 1 mg/mL, and the sample was dried under vacuum and stored at –20 °C. Before the analysis, samples were redissolved in 50 mL of methanol.

### Ultrahigh-performance liquid chromatography–tandem mass spectrometry (UHPLC–MS/MS)

To quantify gangliosides, an UHPLC–MS/MS method was employed in an Agilent 1290 Infinity LC (Agilent, Santa Clara, CA, USA), equipped with an Agilent Eclipse Plus C18 rapid resolution high-definition analytical column (i.d. 2.1 × 100 mm, 1.8 μm, 80 Å) for UHPLC separation. The column was maintained at 50 °C and compounds were eluted using a gradient of 80−90% solvent B from 0 to 0.2 min, then 90–100% for 2 min; 100% of B was maintained for 1 min, and the column was re-equilibrated for a 1-min post run. Solvent A consisted of water and solvent B was 15% isopropyl alcohol in methanol (v/v). Both solvents contained 20 mM ammonium acetate and 0.1% acetic acid. The flow rate was 600 μL/min. Analytes were detected and quantified in an Agilent 6490 triple quadrupole mass spectrometer, operated in the negative electrospray ionization mode. The Agilent Jet Stream electrospray ionization source was set for high sensitivity. The drying gas temperature and flow were 250 °C and 12 L/min, respectively. Sheath gas temperature and flow were 300 °C and 12 L/min, respectively. The nebulizer gas pressure was set at 35 psi, and the capillary voltage was set at 4000 V. The collision cell accelerator voltage was set to 5 V, and the collision energy was optimized on a compound-dependent basis. Nitrogen was used as the collision gas. The resolutions of the Q1 and Q3 quadrupoles were set at the unit resolution. The Agilent Data Acquisition software was used to program the method and for data acquisition. The Agilent MassHunter Qualitative Analysis and Quantitative Analysis Software (v.B.03.01) were used for data processing.

The calibration curves used for quantification were 0, 0.5, 1, 1.5, and 2 mg/L for GD3 and 0, 0.05, 0.1, 0.15, and 0.25 mg/L for GM3. Samples were redissolved in 50 μL of methanol immediately before being injected into the system. The monitored transitions, the optimized collision energies, and retention times are shown in Supplementary Table 1.

Each glycolipid (GM3, GD3) was quantified by summing the area ratio (glycolipid/IS) of all their different species, and interpolating in the calibration curve as shown in the equation:

$$\left[ {{\mathrm{GM}}3} \right] = \left( {\Sigma {\mathrm{GM}}3_{{\mathrm{area}}}{{-a}}} \right){{/b}},$$

where “*a*” is the intercept and “*b*” is the slope of its calibration curve.

### Statistical analysis

Normal data distribution was evaluated by the Kolmogorov–Smirnov test (*p* < 0.05) and homoscedasticity was checked by Levene's test. A one-way analysis of variance (ANOVA) was carried out to evaluate the direct effect of each heat treatment on ganglioside content. A three-way ANOVA was employed to assess the effect heat treatment, storage time, and temperature on glycolipid content. The Tukey test was applied to assess the differences between groups. Version 19.0.1 of the SPSS statistical package for Macintosh (SPSS Inc., Chicago, IL, USA) was used throughout.

The methods were performed in accordance with relevant regulations and guidelines.

### Data availability statement

The data sets generated during and/or analyzed during the current study are not publicly available but can be made available from the corresponding author on reasonable request.

## Electronic supplementary material

Supplementary Table 1

Supplementary Fig 1

Supplementary Fig 2

## Electronic supplementary material

**Supplementary information** accompanies the paper on the *npj Science of Food* website (10.1038/s41538-018-0013-9).
