# Supplementary material for: Application of industrial treatments to donor human milk: influence of pasteurization treatments, storage temperature, and time on human milk gangliosides
Source: NPJ Sci Food. 2018 Mar 13;2:5. doi: 10.1038/s41538-018-0013-9 (PMC6550147; doi:10.1038/s41538-018-0013-9)
Supplement: Supplementary file 1 — Supplementary Table 1 [file 41538_2018_13_MOESM1_ESM.docx]

**Supplementary Table 1** Optimized transitions and collision energy for UHPLC-MS/MS quantification of human milk gangliosides.

| **Transition** | **Ganglioside** | **Collision energy**  **(V)** | **Retention time**  **(min)** |
| --- | --- | --- | --- |
| 1123.7🡪290.1 | GM3 (d32:1) | 50 | 1.53 |
| 1137.7🡪290.1 | GM3 (d33:1) | 50 | 1.64 |
| 1151.7🡪290.1 | GM3 (d34:1) | 51 | 1.75 |
| 1165.7🡪290.1 | GM3 (d35:1) | 52 | 1.85 |
| 1179.7🡪290.1 | GM3 (d36:1) | 53 | 1.96 |
| 1193.7🡪290.1 | GM3 (d37:1) | 54 | 2.07 |
| 1207.7🡪290.1 | GM3 (d38:1) | 54 | 2.25 |
| 1221.7🡪290.1 | GM3 (d39:1) | 55 | 2.35 |
| 1235.7🡪290.1 | GM3 (d40:1) | 56 | 2.38 |
| 1249.7🡪290.1 | GM3 (d41:1) | 57 | 2.48 |
| 1263.7🡪290.1 | GM3 (d42:1) | 57 | 2.63 |
| 1277.7🡪290.1 | GM3 (d43:1) | 58 | 2.71 |
| 706.9🡪290.1 | GD3 (d32:1) | 28 | 1.42 |
| 713.9🡪290.1 | GD3 (d33:1) | 28 | 1.53 |
| 720.9🡪290.1 | GD3 (d34:1) | 29 | 1.62 |
| 727.9🡪290.1 | GD3 (d35:1) | 30 | 1.73 |
| 734.9🡪290.1 | GD3 (d36:1) | 31 | 1.85 |
| 741.9🡪290.1 | GD3 (d37:1) | 31 | 1.94 |
| 748.9🡪290.1 | GD3 (d38:1) | 32 | 2.06 |
| 755.9🡪290.1 | GD3 (d39:1) | 33 | 2.16 |
| 762.9🡪290.1 | GD3 (d40:1) | 33 | 2.26 |
| 769.9🡪290.1 | GD3 (d41:1) | 33 | 2.35 |
| 776.9🡪290.1 | GD3 (d42:1) | 34 | 2.44 |
| 783.9🡪290.1 | GD3 (d43:1) | 35 | 2.52 |
| 1183.3🡪290.1 | Internal standard | 55 | 2.17 |
