# Supplementary figures and images for: Application of industrial treatments to donor human milk: influence of pasteurization treatments, storage temperature, and time on human milk gangliosides
Source: NPJ Sci Food. 2018 Mar 13;2:5. doi: 10.1038/s41538-018-0013-9 (PMC6550147; doi:10.1038/s41538-018-0013-9)

**Supplementary Fig. 1.** Schematic procedure of the experimental design.


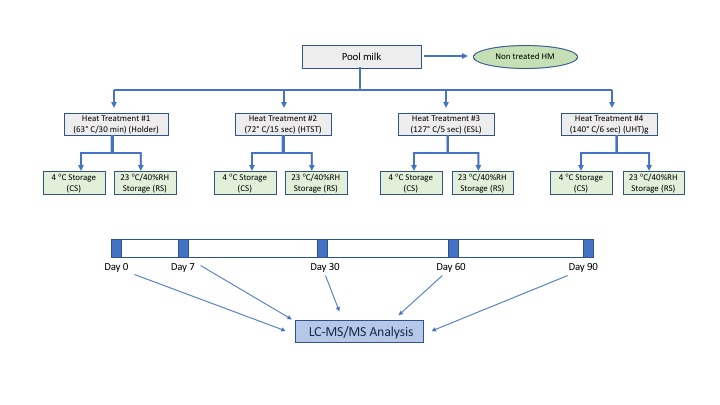

Supplement: Supplementary file 2 — Supplementary Fig 1 [file 41538_2018_13_MOESM2_ESM.docx]
