# Supplementary material for: Application of industrial treatments to donor human milk: influence of pasteurization treatments, storage temperature, and time on human milk gangliosides
Source: NPJ Sci Food. 2018 Mar 13;2:5. doi: 10.1038/s41538-018-0013-9 (PMC6550147; doi:10.1038/s41538-018-0013-9)
Supplement: Supplementary file 3 — Supplementary Fig 2 [file 41538_2018_13_MOESM3_ESM.docx]

**Supplementary Fig.2**. Ganglioside content (mg/L) in human milk before and after being subjected to different heat treatments. Results are expressed as mean ± standard deviation (*n* = 3). Analysis of Variance with HSD-Tukey used as a *post hoc* analysis test (*P* < 0.05). * Indicates statistical differences with non treated milk
